# Women in evolution – highlighting the changing face of evolutionary biology

**DOI:** 10.1111/eva.12343

**Published:** 2015-12-19

**Authors:** Maren Wellenreuther, Sarah Otto

**Affiliations:** ^1^ Department of BiologyUniversity of LundLundSweden; ^2^ Institute for Plant and Food ResearchLundNew Zealand; ^3^ Department of Zoology & Biodiversity Research CentreUniversity of British ColumbiaVancouverBCCanada

**Keywords:** equality, evolutionary biology, gender, women in science

## Abstract

The face of science has changed. Women now feature alongside men at the forefront of many fields, and this is particularly true in evolutionary biology. This special issue celebrates the outstanding achievements and contributions of women in evolutionary biology, by highlighting a sample of their research and accomplishments. In addition to original research contributions, this collection of articles contains personal reflections to provide perspective and advice on succeeding as a woman in science. By showcasing the diversity and research excellence of women and drawing on their experiences, we wish to enhance the visibility of female scientists and provide inspiration as well as role models. These are exciting times for evolutionary biology, and the field is richer and stronger for the diversity of voices contributing to the field.

## Introduction

In the century and a half since Charles Darwin published the Origin of Species, the face of the field has changed dramatically. Emerging from an era with virtually no women practicing as evolutionary biologists, the contributions of women today are many and varied. This special issue highlights the creativity and diversity of women's voices active in evolutionary biology today. No longer are the eminent scientists all male. The leaders of the field are shifting, from a history dominated by male figures – Darwin, Fisher, Haldane, Wright, Dobzhansky, Waddington, Falconer, Stebbins, Mayr, etc. – to a field more balanced by perspectives from both sexes.

While the face of science has been changing, subtle barriers that dissuade women from a career in science remain widespread. Not only is there a paucity of women in leadership positions, but many successful women in science are not as visible as their contributions deserve. An abundance of research demonstrates that having few women represented in science creates a lack of role models to attract and retain young women in scientific professions (e.g. Latu et al. 2013). Women are at the forefront of many scientific advances but their role in driving science forward is not represented equally in courses and textbooks. This was the motivation for Evolutionary Applications to publish this special issue co‐edited by Maren Wellenreuther (Guest editor) and Louis Bernatchez (Editor‐in‐Chief), entitled: Women's contribution to basic and applied evolutionary biology. In particular, we hope that by highlighting the accomplishments of women scientists, this publication may inspire young women scientists, who may be questioning themselves regarding the feasibility and realism of a scientific career, to conclude: ‘Yes we can!’

In this introductory essay, we provide a historical perspective of women in science, followed by an overview of the research contributions in this special issue and closing with a synthesis of the personal reflections contained in each article. In addition, Drs. Rosemary Grant, Mary Jane West‐Eberhard, Josephine Pemberton and Michelle Tseng contributed personal reflections, which are included in this introductory essay. The women invited to contribute to this special issue include both junior and senior researchers in the field, and thus, their reflections also provide a temporal perspective on how barriers have changed. The diversity of their voices provides a great deal of insight into the achievements of these women and their advice for lowering the remaining barriers to women in evolutionary biology.

## A short history of women in science

Evolutionary biology, as a field, developed at a time when women were prohibited from contributing to scientific research. Before the early 19th century, most women were prevented from accessing formal scientific training, so that only women with independent means could pursue scientific study (Orr 2014). This discrimination was founded on centuries of prejudice about women's intellectual abilities. Ironically, these assumptions were often reinforced by pseudoscientific data and paradigms emanating from evolutionary biology. In *The Descent of Man*, with its significant subtitle *Selection in Relation to Sex*, Darwin stated that gender equality was impossible to achieve because the lesser female brain power presented an inescapable consequence of nature (Darwin 1871). Men are simply more intelligent, Darwin argued, because over the millennia their brains became superior due to the need to be effective hunter‐gatherers. Reflecting the beliefs of his time, Darwin wrote ‘The chief distinction in the intellectual powers of the two sexes is shown by man's attaining to a higher eminence in whatever he takes up, than can woman ‐ whether requiring deep thought, reason, or imagination, or merely the use of the senses or the hands’. Scientific arguments were also used to justify why women were expected to stay home. Francis Galton (Darwin's half cousin) and other advocates of positive eugenics had, for example, warned that if women of distinguished pedigree turn their back on being nurturing mothers, then the quality of the next generation would decline (Fara 2015). Consequently, it was expected that women divert all their energy into childrearing and not indulge themselves in education or employment. While suffragists rallied for women's rights and counterargued that evolutionary principles emphasize the fundamental similarities among members of a species, their opponents emphasized Darwin's ideas on the role of sexual selection, which supported the cultural prejudices of female intellectual inferiority (Richards 1997).

The laws that prohibited women from seeking education in Europe and North America were gradually overturned during the mid‐19th century, with the opening of the first women's colleges (Etzkowitz et al. 2000). While these colleges granted women access to both education and employment, they unfortunately also applied strict employment rules, which came at a considerable personal price: all faculty had to remain unmarried, a practice that in some parts of the Western World continued even through World War I and II (Barnett and Sabattini 2009). Employment chances of women were low and reduced by the requirement by many colleges that faculty hold a PhD degree, despite the fact that most European and North American universities refused to allow women into their graduate programmes (Barnett and Sabattini 2009). In the 1890s, a small number of institutions allowed women to matriculate into advanced degree programmes, but most institutions were slow to follow this lead.

It was only in the second half of the 20th century that women gained access to graduate education in large numbers and had the potential to become professors. Some traditional all‐male colleges circumvented the need to grant official access to women by opening all‐female sister institutions that coexisted alongside the prestigious all‐male colleges. Harvard, for example the oldest institution of higher education in the United States, was founded in 1836 to educate an all‐male clergy. Nevertheless, women showed a persistent interest to attend courses. When lectures at Harvard were opened to the public in 1863, women immediately flocked to attend (Ulrich 2004). Indeed, Harvard proved so popular with women that by 1870 they presented the majority of all students (Ulrich 2004). Public access to the open lectures was suspended when Harvard established its own Graduate Department in 1872 (Ulrich 2004). This move was met by considerable resistance, which Harvard solved by simply opening a sister institution called ‘The Society for the Collegiate Instruction of Women’ in 1879 (later to become Radcliffe College). This allowed women to receive instruction from Harvard professors who were willing to earn extra income by teaching their courses twice, once for men and once for women. It was only in 1963 that Radcliffe Graduate School merged with Harvard Graduate School, after having awarded 750 PhD degrees (Horowitz 1986). Likewise, Princeton only became co‐educational in 1969 (Selden 2000). An early attempt to establish a parallel institute for women (the Evelyn College for Women) closed in 1897, ten years after its founding, due to financial problems and a lack of support from Princeton (Selden 2000).

In addition to a lack of access, the scientific achievements of women have traditionally been underrecognized. Women have historically been limited to secondary roles in science production and authorship, as translators, illustrators and popularizers of science, and these by virtue of marriage or kinship relations with eminent male scientists (Orr 2014). As a result, women were all too often portrayed as ‘volunteer’ faculty members, with credit for their significant discoveries assigned to male colleagues. Esther Lederberg (1922–2006), a microbiologist, conducted ground‐breaking research in the field of genetics, most notably on bacteriophages. She discovered lambda phage, a virus that infects *E. coli* bacteria and published the first report of this in Microbial Genetics Bulletin (Lederberg 1950). Her work helped her husband, Joshua Lederberg, win a Nobel prize in 1958, which he shared with Edward Tatum and George Beadle (Harvey 2012). Likewise, Rosalind Franklin (1920–1958), a pioneering X‐ray crystallographer, developed images of DNA molecules that were critical to deciphering its structure – one of the biggest and most important scientific breakthroughs of the 20th century. Recognition for her contribution to the discovery of the DNA structure remained limited during her lifetime (Jones and Hawkins 2014; Orr 2014).

Even less attention has been given to the discovery of Dr. Marthe Gautier, who first identified aneuploidy as the cause of Down syndrome (Pain 2014). As a young French researcher, she received a prestigious scholarship in 1955 to undergo medical training at the paediatric cardiology unit at Harvard to examine cell cultures. Upon her return to work in Paris in 1956, she became involved in genetic research on human diseases. In the same year, the human chromosome number was corrected to 46 (Harper 2006), allowing researchers for the first time to detect chromosomal abnormalities (Gartler 2006). The head of the paediatric unit, Dr. Raymond Turpin, was interested in the possibility that Down syndrome was caused by a chromosomal abnormality and provided Gautier with a tissue sample from a patient and a disused laboratory equipped with a poor‐quality microscope (and no funding). Despite these inauspicious conditions, Gautier discovered that the karyotype from the Down syndrome patient contained an extra chromosome, but she was unable to establish which chromosome was involved using her microscope (Gautier and Harper 2009). She entrusted the slides to Dr. Jérôme Lejeune, who established the karyotype using a higher quality photomicroscope and announced at a conference that he had discovered the cause of Down syndrome. He went on to publish the story with Gautier's name as middle author (Lejeune et al. 1959) – a paper she did not get to see and knew nothing about until the day before publication (Gautier and Harper 2009).

‘La découverte de la trisomie n'ayant pu être faite sans les contributions essentielles de Raymond Turpin et Marthe Gautier[;] il est regrettable que leurs noms n'aient pas été systématiquement associés à cette découverte tant dans la communication que dans l'attribution de divers honneurs’.

[The discovery of trisomy would not have been possible without the essential contributions of Raymond Turpin and Marthe Gautier[;] it is regrettable that their names have not been systematically associated with the discovery in both the communication and attribution of various honors.]

– Ethics Committee, French Institute of Health and Medical Research (2014) 1

Recognition of women's achievements through membership in academies was also restricted. Indeed, three of the most prestigious scientific societies, the *Royal Society of London* (the oldest continuous society of science, founded 1660), the Parisian *Académie royale des Sciences* (founded 1666) and the *Akademie der Wissenschaften* in Berlin (founded 1700), did not permit women to become members for almost 300 years following their establishment (Schiebinger 1993). Marie Curie, probably the best known female scientist, who earned two Nobel Prizes, was turned down for membership of the prestigious *Académie royale des Sciences* in 1911, the very year she went on to win her second Nobel Prize. In fact, it was only in 1979 that the first woman, the physicist and mathematician Yvonne Choquet‐Bruhat, was elected as a fellow (Schiebinger 1993). The mathematician Hertha Ayrton (1854–1923) became the first woman nominated as fellow of the *Royal Society of London* in 1902, but she was refused this distinction on grounds that she was married (Mason 1991; Fara 2015). More than four decades were to elapse before the next women were nominated. Finally, in 1945, Kathleen Lonsdale (1903–1971), an early pioneer of X‐ray crystallography, along with microbiologist Marjory Stephenson (1885–1948) were the first women to be admitted as fellows (Glazer 2015). It took the German *Akademie der Wissenschaften* even longer to open its doors to women. Only in 1964 was Elisabeth Welskopf‐Henrich (1901–1979), a participant in the resistance during World War II and professor of history, elected as its first full fellow (Wobbe 2002).

While inequalities in these three academies were strong and prevented the admission of women for over 300 years, gender disparities exist even nowadays in many of the scientific societies and academies. For example, the National Academy of Sciences (NAS) in the United States was founded in 1863, yet despite this comparably much younger age, the membership remains heavily skewed towards men. The total number of active and emeritus members is 2113, of which a mere 219 are women (similar numbers hold if we include foreign associates: 2508 total versus 251 women^2^). The Association for Women in Science (AWIS) has examined the NAS membership distribution over the last 20 years and found that for many years women were underrepresented relative to the available pool of Ph.D. holders worthy of consideration.^3^ An even stronger division can be seen if we look at the gender distribution of Nobel Prize laureates. The Nobel Prize is by many regarded as the most prestigious award given for intellectual achievement in the world, yet between 1901 and 2014 only 3% of all Nobel awardees in Medicine, Physics or Chemistry have been women.^4^

When looking more specifically at the field of evolutionary biology, these biases persist. The Society for the Study of Evolution has one annual prize to recognize the accomplishments of outstanding young evolutionary biologists called the Theodosius Dobzhansky Prize. The prize was established in 1981 and has only been awarded to a woman four times since its establishment.^5^ A similarly distorted sex ratio is evident when looking at presenters at evolutionary biology meetings. A recent study analysed data on the sex ratio of presenters from the European Society for Evolutionary Biology (ESEB) biannual congress 2011 and found that women were underrepresented among invited speakers at symposia (15% women) compared to all presenters (46%), regular oral presenters (41%) and plenary speakers (25%) (Schroeder et al. 2013). This underrepresentation of women was found to be partly attributable to a larger proportion of women, than men, declining invitations, yet the reasons for this are not known. A possible cause for the higher decline of invitations by women may be related to family responsibilities, such as child rearing or looking after elderly people, which are both activities that are predominately carried out by women (Namrata et al. 2005). Similarly, managing domestic responsibilities poses a challenge for research projects that involve extensive travel for fieldwork.

The representation of women in science improved considerably in the second half of the 20th century (England and Li 2006) and continues to do so. Particularly, notable pioneering women in evolutionary biology emerged during this time, including Barbara McClintock, Tomoko Ohta, Mary Leakey, Lynn Margulis and Margaret Kidwell. Over this period, the numbers of women and men pursuing science degrees have converged and are now nearly equal. Despite a significant increase in women graduating in science degrees, the proportion of women in science leadership or as research professionals decreases dramatically with seniority. In the United States, for example, even though women earned approximately half of all science bachelor (55.6%), master (54.0%) and doctoral (46.7%) degrees in 2010, only 34.5% of all professors are women, and this representation declines further to 21% among full professors (NSF 2013). While the number of women advancing through the ranks has risen as more and more women earn graduate degrees, the percentage of women seen at the more senior levels is not mirroring the increase seen at the graduate level. The ‘leaky pipeline’ is particularly acute at two stages: as women enter academic positions and as women reach the highest echelons in academia (the equivalent of Full Professorship) (Council of Canadian Academies 2012). As concluded by a recent report of the Council of Canadian Academies (2012), ‘these data indicate that time alone will probably not be enough to balance the proportion of women and men at the highest levels of academia’.

Among the challenges women face advancing through careers in science, one factor is systematic bias in evaluating the sexes (Reuben et al. 2014; Leslie et al. 2015). Research shows that both men and women tend to overrate men and underrate women in competence, particularly when women are in nontraditional fields such as science, and that this bias occurs unconsciously (Zuk and Rosenqvist 2005). For example, a 2012 study showed that both men and women tend to undervalue women candidates for a research technician position, even when the applications are identical except for the name of the candidate (Moss‐Racusin et al. 2012). Empirical studies of gender equality document a broad bias against representation of women in conference presentations (Schroeder et al. 2013; Jones et al. 2014), scholarly authorship (West et al. 2013) and invited journal articles (Conley and Stadmark 2012). Additionally, research has demonstrated that more stringent criteria are used to measure women's qualifications in grant evaluation panels (Wennerås and Wold 1997; Reuben et al. 2014; Leslie et al. 2015) and that women receive smaller grants and fewer nominations for awards (Cho et al. 2014).

As an example, Tomoko Ohta – best known for her development of the nearly neutral theory – reflected on her initial struggles against implicit bias when first working in the laboratory of Motoo Kimura. ‘*Kimura was a typical Japanese man of his time, who regarded women's scientific activities as insignificant. After two years or so, I had convinced him that I should continue to do research*’ (Ohta 2012).

On top of the systematic bias and more stringent criteria lies outright sexual harassment. Studies in the United States and Europe demonstrate that a considerable number of women in STEM had to face sexual harassment at work. For example, Sonnert and James Holton (1995) surveyed 191 female fellowship recipients in the United States and found that 12% of them had been sexually harassed during their graduate school or early professional experience. Likewise, a study on Finnish University academic staff found that about 7% of employees had suffered sexual harassment, 78% of whom were women (Mankkinen 1999). Based on an internet survey of scientists’ experiences at field sites, Clancy et al. (2014) found that gender was a significant predictor of having personally experienced sexual harassment, with women respondents being 3.5 times more likely to have experienced sexual harassment than men. These studies also highlight that sexual harassment may take multiple and diverse forms, from serious harassment to the overemphasis of sexual roles, and can provoke a deep feeling of isolation and professional discouragement.

Another contributing factor is that the ‘paucity of women in leadership positions makes it difficult for other women to envision themselves as leaders’ (Council of Canadian Academies 2012). To understand that science careers are realistic options, women need to see the evidence that those they identify with, people like them, can and do succeed (Latu et al. 2013). Seeing women with children in science leadership positions, and how they balance their careers and personal lives, is particularly important for the younger generation of women who struggle to imagine how to combine family life with a career in science (Shen 2013). They also need to know that the people around them see a career in science as a valid choice for them.

## Revising perceptions of women in evolutionary biology

A powerful way to counteract this skewed and biased representation of women in science is to showcase their contributions openly (Jones and Hawkins 2014). By requesting articles from women in evolutionary biology, the goal of this special issue is to highlight the diversity of research performed by women in the field today. Fortunately, nowadays there are so many women succeeding in evolutionary biology that it would be impossible to feature all of their research, but we asked for contributions from a representative sample of women, at various stages in their careers, working in a variety of subfields, and across a diversity of countries.

The authors were asked to contribute both research articles and personal reflections (see next section). The research they describe reflects the strength and diversity of evolutionary biology today, regardless of gender. The articles in this special issue span the breadth of areas in evolutionary biology and touch upon many of the hottest topics, including sex chromosome evolution (Charlesworth 2016), sexual selection (Servedio 2016; Wellenreuther and Sánchez‐Guillén 2016), behavioural evolution (Aubin‐Horth 2016; Charmantier et al. 2016), evolution of cooperation (Aktipis 2016), diversification and speciation (Charmantier et al. 2016; Johannesson 2016; Qvarnström et al. 2016; Servedio 2016; Wellenreuther and Sánchez‐Guillén 2016), host–parasite interactions (Leftwich et al. 2016; Myers and Cory 2016), evolutionary change accompanying invasion (Aktipis 2016; Gillespie 2016; Haig et al. 2016; Lee 2016; Olivieri et al. 2016; Sork 2016), genomics and genome evolution (Aitken and Bemmels 2016; Charlesworth 2016; Charmantier et al. 2016; Johannesson 2016; Sork 2016), and climate change (Aitken and Bemmels 2016; Qvarnström et al. 2016). Although there are undoubtedly some areas of evolutionary biology that have further than others to go to achieve gender equality, women are strong contributors to all subfields, from theory to genomics, from behaviour to systematics.

Among the research contributions, some articles summarize classic work by the authors that has shaped our understanding of the field. Charlesworth (2016) describes her theoretical research to understand when and how nonrecombining gene complexes (‘supergenes’) are formed, as well as empirical efforts to determine the relevance of supergenes (e.g. to sex chromosomes, mimicry and distyly). Myers and Cory (2016) describe long‐term studies to understand how insect population dynamics are shaped by pathogens, particularly the cyclic outbreaks seen in species such as western tent caterpillars. Gillespie (2016) describes her now‐classic work in the Hawaiian archipelago on spider diversification, which spans a range of low levels of ‘nonadaptive radiation’ in some groups (without ecological diversification) to high levels of adaptive radiation (new species formation with ecological diversification). Aubin‐Horth (2016) describes the exciting emergence of ecological genomics as a subfield, which has opened up the black box linking molecules and environmental cues to phenotypic and behavioural variation, focusing on her work on salmonid alternative reproductive tactics.

The papers also explore new topics and altered perspectives. Qvarnström et al. (2016), for example, investigate the possibility that adaptation to different climates may drive speciation, even if this divergence is difficult to detect morphologically. Servedio (2016), in her recent theoretical work, has explored the interplay between sexual selection and speciation, yielding many counterintuitive results that challenge our understanding of these processes (e.g. finding that stronger sexual selection can hinder rather than promote divergence between populations and that mating preferences can reduce local adaptation of traits).

Several research articles delve into the evolutionary forces acting in particular species, describing the evolutionary insights that come from an in‐depth understanding of a group of organisms. Wellenreuther and Sánchez‐Guillén (2016) describe the laboratory's research on damselflies, which leads them to conclude that divergence in reproductive traits, not ecological differences, has driven speciation in this group. Johannesson (2016) describes her work to rationalize the messy taxonomic treatment of *Littorina saxatilis* snails, while previously split into many species due to shell polymorphisms that arise between crab‐rich and wave‐swept microenvironments, her work has shown that these ecotypes arise repeatedly but are likely prevented from speciating due to gene flow and the high fitness of hybrids in intermediate environments. Penczykowski et al. describe how their work in various host–parasite systems is revealing the spatial and temporal scales at which co‐evolutionary (mal)adaptation occurs and the insights this yields about the underlying dynamics (e.g. fluctuating selection versus arms race). Charmantier et al. (2016) describe over 40 years of monitoring blue tits (*Cyanistes caeruleus)* that has revealed the strong connection between habitat heterogeneity and diversity in life‐history, behavioural and reproductive strategies.

Women are also at the forefront of evolutionary applications. Aitken and Bemmels (2016) describe work to improve reforestation practices, switching from current ‘local is best’ reseeding strategies towards more diversified stocks (‘assisted gene flow’), incorporating seeds that are adapted to warmer environments to better protect future forests. Chapman and colleagues review efforts to improve biocontrol of insects, including work in her laboratory to use antagonistic seminal fluid proteins as a means of population control (Leftwich et al. 2016). From their decades of work on endemic plants, Olivieri et al. (2016) conclude that conserving the landscape that generates genetic diversity is critical for the maintenance of evolutionary potential, a factor that must be incorporated into management strategies to better protect extant biodiversity and biodiversification. Haig and colleagues review their efforts to conserve species at risk via the application of genetics to taxonomic delimitation, to determine landscape use and migratory connections, and to guide breeding programmes for recovery from severe bottlenecks (Haig et al. 2016). Applied research by Lee's group is investigating evolutionary changes that accompany the invasive spread of a species by comparing parallel invasions into freshwater of the copepod *Eurytemora affinis* (Lee 2016).

### Learning from personal reflections

**Figure 1:**
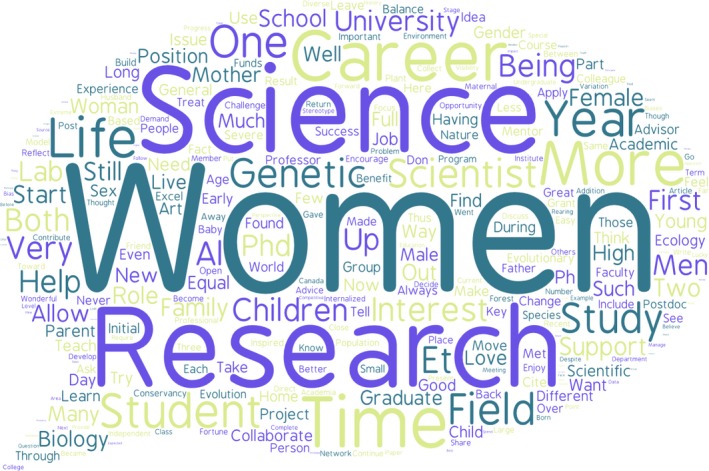
A word cloud of the personal reflections in this volume (tagul.com).

In addition to requesting research articles, the editors of this issue asked women to contribute a section or box describing personal reflections (see Fig. 1). The content was left open: ‘your personal opinions or experiences regarding any professional aspects of the life of female evolutionary biologists that you would feel relevant to mention’. While not all women approached could contribute full‐length research articles, many were keen to support this special issue in other ways. Three contributed their personal reflections to this chapter (Box 1: Rosemary Grant; Box 2: Mary Jane West‐Eberhard; Box 3: Josephine Pemberton). We also requested the personal reflections of Michelle Tseng, who conceptualized, launched and managed *Evolutionary Applications* from 2008 to 2013 (Box 4).

Box 1 **Personal reflections of Rosemary Grant, Princeton University, USA.**

I was born in 1936 in a small, coastal village, in the Lake District of North West England. It is an area of carboniferous limestone fossil‐rich cliffs, backed by wooded valleys and high grass fells where rare species of butterflies and plants can be found.

Even before I knew the word biology I was fascinated by the diversity of organisms around me. My parents told me that some of the fossils I had found were of plants and animals that were now extinct, and this added to my excitement and curiosity. A delightfully perceptive old gardener, (Jerry) Jeremiah Swindlehurst, who had never been to school but taught himself to read and write, was a constant inspiration and source of local biological wisdom. When I was twelve my Father, who was a physician, suggested I should read Darwin's ‘Origin of Species’.

In my teens I thought that genetics would provide some basic understanding of this variation, and I desperately wanted to study genetics at Edinburgh University. The head mistress of the all‐girl's boarding school I attended dissuaded me from taking all the necessary university entrance examinations, saying: ‘A girl with two brothers should not go to university’. This was the norm in 1950s Britain. When it was time to take my Scottish higher leaving certificate in 1954, I had mumps with pancreatic complication…leaning over my bed the mistress's words were: ‘It is God's will you should not go to university’.

I left school, determined rather than deterred, took a job, followed a correspondence course and applied on my own to university. Although it did not seem so at the time, this course must have immeasurably enhanced my chance of getting good grades in the examinations, because the school I had attended was academically weak.

My good fortune in entering Edinburgh University was enhanced when in my third year I was accepted into the genetics diploma course, consisting of a very small group of national and international students studying under Professor C.H. Waddington. My mother and Jerry gave me the love of nature, my father showed me how to study it, and Conrad Waddington, Douglas Falconer and Charlotte Auerbach inspired me to be a scientist.

My first project was an undergraduate thesis on soil amoebae. Among many identical looking amoebae living in the soil, one was thought to be pathogenic. My genetics training had introduced me to the possible importance of differences in cell surface proteins. I made antibodies (from rabbits) to the pathogenic one that had been collected from mammalian tissue. I then dropped serum containing these antibodies onto slides with soil amoebae. It worked! The dangerous amoebae clumped together; the benign ones were unaffected. Spurred on by this small success, I wanted to tackle a much larger question: How and why do populations of organisms diverge to the point of becoming different lineages? At the time, I thought that char, landlocked in fjords of known ages in Iceland, would be perfect to study this. Before I started my PhD, I had the opportunity to teach embryology for initially one year, 1960–1961, at UBC in Canada. Here I met Peter. It was thrilling to find we had similar interests and similar goals. At that time, Peter approached the same questions from an ecologist's viewpoint, while I approached them more from a geneticist's point of view and the synergism was electrifying. We were married in January 1962.

With two small children and in those days a lack of good day care facilities in Montreal, I stayed at home until they went to school. One day a week, on Mondays, I had a babysitter, and instead of spending that day catching up on household chores, I spent it in the McGill library catching up on research articles. This put me in a good position when I finally was able to return to full‐time research, slowly via an intermediate step of high school teaching in Montreal.

Our two children always accompanied us while doing fieldwork on Darwin's finches in the Galápagos. We homeschooled them during the lunch hours. One on one is more effective and creative than a classroom, and they were always ahead of their class on return to school. Looking back neither remembers receiving ‘lessons’ on the islands. Now they are mother's themselves and both say that taking them into the field was the best thing we ever did.

We moved to the University of Michigan in Ann Arbor in 1978, and I returned to full‐time research and worked on my PhD project on the Large Cactus Finch on Genovesa Island in the Galápagos. Staffan Ulfstrand at the University of Uppsala in Sweden was my supervisor. He was invaluable, allowing me flexibility in pursuing my own research interests and at the same time immensely stimulating with his provocative questions.

Thus, I entered a fully professional career through false starts (at high school) that did not put me off, with a hiatus (childrearing), which I enjoyed, and a husband (Peter) who gave me immense support when the time came to get a PhD and return to full‐time research and teaching.

**Research**

How do new species form? How do populations of organisms diverge and become different enough that they no longer interbreed, or do so rarely? On the Galápagos Islands, we tackled the problem of species formation from many different angles, examining the ecology, behaviour and genetics.

Climatic swings caused by El Nino Southern Oscillation phenomenon brought years of torrential rain interspersed with drought years, and it was these drought years when 80–90% of individuals died that allowed us to measure the strength of selection and evolutionary responses to this selection in the next generation.

An unexpected finding was rare genetic exchange between closely related species of similar body size. In Darwin's finches, the premating barrier is based on species‐specific song learned from the father in association with parental morphology during a brief receptive period early in life. Being based on learning, this barrier is vulnerable to disruption. On rare occasions, a young bird learns the song of another species, as a result of nest take over, or death of the father, and this can lead to hybridization. Whether or not this hybrid survives depends on ecological conditions, and on whether there is appropriate food for birds of intermediate beak size. When suitable food is available, backcrossing to one or other parental species according to song type leads to genetic exchange between species. We were fortunate to witness an unprecedentedly severe El Nino in 1983 that changed the ecological conditions of the island to one that allowed the survival of hybrids. It led to genetic exchange between the medium ground finch (*Geospiza fortis*) and cactus finch (*G. scandens*) populations over the next 30 years. Although hybridization was never more than 1–2% of any breeding attempt, over time the genetic and phenotypic variation of both populations was increased to a measurable and noticeable extent. This is a situation that allows a rapid evolutionary response to natural selection events in a new environment.

The most unanticipated finding in our many years of research was the formation of a new lineage as a consequence of genetic exchange, which we followed from its inception over the next six generations. His descendants formed a population that was unique in song and morphology and became reproductively isolated from all other finch species on the island.

Having found that genetic exchange played a key role in forming a new lineage in contemporary time we were gratified to learn of genetic exchange throughout the whole of the Darwin's Finch radiation. This discovery, published this year, came from analyses of genome sequences by Leif Andersson and his group from Uppsala, using blood samples that we had collected. It suggests that the fuelling of genetic variation through hybridization has contributed not only to a new lineage in contemporary time but also to the formation of new phenotypes and the development of new species in the past.

**Advice to women scientists today**

In the 1950s, two pieces of advice were given to me. First, never use your full name, only initials on examinations. Second, a male professor with daughters and no sons is often the most supportive! Today, I would say to all young scientists, follow your passion and the direction that most interests you. Value your exceptions, be open to alternative explanations and do not hunt by expectation. The road will not be smooth, but there will be magic in it.

Box 2 **Personal reflections of Mary Jane West‐Eberhard, Smithsonian Tropical Research Institute, Costa Rica.**

Every scientist seems to have definite ideas about women in science based on personal impressions. Here, I express some of my own, knowing that real data would be better but unable to resist the opportunity to say what I think: that a woman scientist who wishes to have children and is conscientious about their care still faces special challenges if she also wishes to be competitive in her career and enjoy the excitement of a life in science – of creative discovery, of international travel and collaborations, and of teaching and writing that may be of lasting benefit to humanity.

In my own reflections on women in science, one not‐very‐surprising generalization leaps forth: the career trajectories of women are often different from those of men. The reason of course is that the child‐rearing role of women and their shorter reproductive lifespan more often pull them away from an ascendant career. Sexual asymmetries in reproductive lifespan and parental investment will continue to affect humans, at least in the foreseeable future, along with sexual asymmetries in parental role: certain evolved morphological, physiological and psychological characteristics enhance the ability of most mothers to sense and respond to the needs of offspring, to the benefit of all concerned (offspring and both parents). Despite great variation in the parental commitments, inclinations and abilities of men and women alike, the evolved sex asymmetries are biological facts that demand special accommodation if a society aspires to take advantage of both the maternal and the scientific abilities of women. Fortunately, reproductive biology need not limit intellectual destiny if social arrangements resolutely accommodate both.

The *historical* result of the sex differences just mentioned is that employment policies reflect male life‐history trajectories. So a man's career is automatically more easily harmonized with family life, whereas each woman scientist has to find some idiosyncratic pathway to having both a career and a family. A particular woman's pathway depends on individual variables, such as her career progress relative to her age, attitudes of family and teachers towards an unusual female role, whether or not she falls in love and when, or decides to have children and when, or comes from a culture that condones work by women outside of the home. This background of asymmetrical conditions and adjustments has not changed much through the four generations familiar to me personally – my grandmother's, my mother's, my own and my daughter's.

As a result of these fundamental biological and social asymmetries, women who have been successful in demanding fields such as science have always been some combination of unusually *determined, motivated, persistent, stubborn, rebellious, irrepressible or independent‐minded* – qualities also heightened in professionally successful men but not always seen as virtues in women. One sign of progress is that women scientists now do not have to endure those adjectives so often used as euphemisms for *pushy, strident, aggressive, selfish, obnoxious, mannish, unbecoming and less mentionable words*. Early in my own career – in the 1960s – I felt that avoiding such epithets required a sufficiently feminine manner of behaviour and dress to mitigate the qualities of assertiveness and independence needed to compete successfully as a scientist, while at the same time avoiding stereotypical female behaviours that could lead to being dismissed as ‘just a woman’. I had some rules. For example, do not keep your notes for a plenary lecture in a dainty ladies purse and pull them out at the podium (as I once saw a woman do). To get an idea of the image of femininity that permeated the atmosphere of my generation where I lived in the Midwestern USA, and more broadly including in the world of science, look at some romantic movies from the 1950s or TV comedies like ‘George Burns and Gracie Allen’ and ‘I love Lucy’ – in which female eccentricity and independent mindedness are portrayed as comical frivolity that can be harmonized with domesticity only by a forbearing husband.

It is interesting to ask particular women why they went into science despite the challenges of doing it. I once heard someone say that ‘men branch out, but one woman leads to another’. If you ask a successful woman scientist what her mother did, you often get a revealing reply, and the replies have changed over time. Women of my grandmother's generation would often describe a mother with strong character and an independent mind, not often a career. For my mother's generation, the answer often referred to a mother's hobby, like a love of botany or natural history. For my generation – growing up in the 1950s (I was born in 1941) – the answer more commonly described a mother's career outside of the home, such as primary school teaching, or work as a nurse or a secretary, but still careers oriented towards children or support rather than leadership, and only very rarely a career in science or the professions that would require long training and long hours away from home, except as a collaborating wife. Still, the generational shift in the replies I have heard suggest progress for women in science. My own genealogy has a good dose of the right stuff for motivating generational change in the right direction: my mother was a schoolteacher with a master's degree in primary education and was the first of her family to attend college. Before her was my favourite grandmother, a tough and stubborn farm woman known for working and cussing like a man, whose own mother (my great‐grandmother) was so independent‐minded that, even though mild mannered and kind‐hearted, she divorced an abusive husband, an act so rebellious and unusual for her time that it was never mentioned even within the family when I was small, except in hushed tones.

Despite obvious progress, especially in the increased collaboration of partners in child rearing and ample parental leave in a few countries, the central problem for women in science – still incompletely solved – is how to harmonize relationships and family life with a full‐blown scientific career. This largely boils down to childcare. Even today woman scientists are obliged to adopt extreme solutions to this everyday human task. Some of them decide not to have children. Others postpone motherhood until they feel established in their careers or postpone their careers until they feel their children are sufficiently launched. Others have a family fortune or a wealthy husband that allows them to hire expert help; others spend most of what they earn to obtain it. Some decide that children are so important and enjoyable that they drop out of science and then find that they cannot readily get back in. Others, with unusual stamina and determination, become ‘superwomen’ who seem able to do everything at once. My own extreme solution was to leave the United States to live in Latin America. Before leaving, I cleverly married Bill Eberhard, a prolific evolutionary biologist with wide interests that overlap broadly with mine – in effect, a portable home biology department and my closest scientific colleague, who also loves kids (we have three). And then, I serendipitously got a wonderful job with the Smithsonian Tropical Research Institute which allowed doing research literally in our back yard, in countries (Colombia and Costa Rica) where we could afford a full‐time helper for household chores and babysitting.

It should not be necessary for woman scientists to adopt any of these extreme solutions, or for their partners to be unusually committed to childcare. I hope that the situation for women will continue to change until there is no longer a need for exceptional arrangements for living a balanced life. No issue is more important now than working towards a true accommodation of the child‐bearing and child‐rearing biology of women and men, through, for instance, ample parental leave, temporarily part‐time early‐career appointments that still get full respect, policies that permit ample participation of supportive partners, reassignment of academic duties to allow more work at home and a smoothing of the on‐ramp to a full life of science after the temporary off‐ramp required by maternity and family life. Scandinavian‐style free public day care is only a partial solution because even at its best it does not achieve the goal of allowing, and *encouraging*, parents to spend time with their children, including consideration of the long hours of overtime work currently required to sustain a career in science.

I realize that women face barriers not *seen* as child‐rearing problems, such as differences in classroom treatment, hiring, salaries and promotions. But I believe that family demands are the root causes of the irregular career paths of women and of the generalized biases that can accumulate during a lifetime to reduce the likelihood of a woman being in the top echelons of science. So I believe that measures that help to harmonize pregnancy and child rearing with scientific careers are fundamental for the progress of women in science, whether they have children or not.

Box 3 **Personal Reflections of Josephine Pemberton, University of Edinburgh, UK.**

Raising children has not happened for me. Instead, I have gone through a scientific career at a pace where I feel very well rewarded and have spent the last 5 years as head of an institute containing about 32 principal investigators (i.e. faculty and independent fellows), as well as being heavily involved in running two long‐term studies of wild mammals on Scottish islands (red deer on the Isle of Rum and Soay sheep on St. Kilda).

I was born in 1957 and brought up in Wimbledon, London. Rearing tadpoles through to frogs in a tank in my bedroom is one of my earliest memories, and there followed many pets including fish of all varieties, terrapins, tortoises, budgies, cockatiels and guinea pigs as well as the much‐loved family dog. I was an early member of the Young Ornithologist's Club, the youth wing of the UK's largest bird conservation charity, the Royal Society for the Protection of Birds. I was animal‐mad, although perhaps unusually, my equestrian phase was only brief. Wimbledon High School had a nature study prize, named after a previous pupil called Jennifer Crafter. My dismay on discovering that the main exercise set involved observing a plantain plant through the reproductive season was intense, but I won it anyway. With five like‐minded school friends, I formed the Wimbledon Society for the Protection of Nature (WSPN) which was an excuse for a weekly postschool tea party.

This early life was disrupted by my being sent to an all‐girl boarding school for 6.5 years. This forced me along academically so well that I won a scholarship to read Zoology at Oxford for which I am profoundly grateful, but I suspect it also stunted my social development. At Oxford, I lived in college (a reflection of my lack of social development?). I worked hard and got stressed: who would not with a biochemistry tutor who reminded one that all previous holders of this particular scholarship got first class degrees? I did not, which I hope has made it easier for my successors! Summer vacation volunteering in Kenya got me hooked on large mammals, and a PhD opportunity in Reading pushed me in the direction of genetics. The principle finding of my thesis was that the fallow deer (an abundant introduced species in the UK) lacks genetic variation as studied by allozyme electrophoresis.

I had always been interested in the opportunities offered by individual‐based studies, because of the potential to understand sources of variation in survival and breeding success. Since hearing about it as an undergraduate, I had been interested in joining the red deer study on Rum. Starting in 1972, the remarkable Fiona Guinness had lived very remotely on Rum, recognizing each individual deer in the study area from its facial and other features (we use tags and collars these days) and following their lives in extraordinary detail. Tim Clutton‐Brock realized the potential of such data to answer many questions in ecology and behaviour, wrote many key papers and raised funds for continued data collection and analysis, the latter being mostly performed by Steve Albon. I was lucky to be funded to investigate genetic variation in the deer, at first using allozyme electrophoresis. Alec Jeffreys’ discovery of the class of loci called minisatellites, which enabled DNA fingerprinting to identify parentage in natural populations, provided me with a key break, and ever since I have been engaged in reconstructing pedigrees and investigating sources of variation in traits, including fitness components, in both the red deer, and since the inception of the sister project in 1985, the Soay sheep. After a very happy nine years of postdoc work in London and Cambridge, I won a lectureship in Edinburgh and have been here ever since.

My colleagues and I in Edinburgh now run both the deer and Soay sheep projects, ably assisted by a wide range of collaborators at other universities. Like the study of Rosemary and Peter Grant in the Galapagos, these are long‐term studies straddling the disciplines of ecology and evolution, in which the data sets become more and more valuable as complete individual life histories accumulate and can be interrogated to answer different questions. Indeed, we share our data with many people who suggest new data analyses that we do not plan to do. The major challenge with such projects is, of course, to keep them running in a funding landscape which is hugely competitive and where grants last for just three or at best 5 years. It is a constant exercise in scanning the horizon for ideas.

With regard to women in science, I have two reflections. First, as well as addressing the difficulties women in science face, I think we should be on a mission to identify what advantages there are to the progress of science to have more women in the system, especially at senior levels. In business, studies have found a correlation between having women on company boards and company profits (Luckerath‐Rovers 2013). Whether this is a causal relationship and why it occurs are both unclear. Nevertheless, the internet is full of evidence of initiatives to increase representation of women on boards, and progress seems to be fast in some cases (e.g. Women on Boards: Davies Review Annual Report 2015). What about women in science? Strictly anecdotally, my impression is we are more predisposed to try and help with an identified problem; we treat students more sympathetically; we are more likely to be cautious about publishing an unexpected result; and we are perhaps more interested in a long game than in short‐term glamour. I have not read much research on any of this, although there is some evidence that women are underrepresented in cases of scientific misconduct (Fang et al. 2013). Are these characteristics good for science? Clearly, a mix of talent is required, but they certainly make running a department much easier and my bet is that they are.

My other reflection is about promotion. The criteria for promotion in UK universities are strongly biased towards research prowess, for example rewarding high profile papers and grant acquisition more than teaching success (although emphasis is changing towards the latter). As discussed in Mary Jane West‐Eberhard's piece, women traverse this process at widely different rates, depending on their investment in family life. This sets up a sex difference in the underlying cause of status variation. Men by and large hold positions reflecting their research prowess. Women hold positions reflecting their maternal status (I exaggerate somewhat, of course) because women engaged in childcare cannot simultaneously fulfil their full potential as researchers. It is surely hugely frustrating for women to see themselves being overtaken, and as a head of institute, I have not enjoyed seeing this happen. I do not necessarily think it would be fair to either men or childless women to change the promotion system (beyond incorporating far more reward for good teaching). Rather, we should continuously remind ourselves that having children is a career in itself. I am always incredibly impressed by anyone who holds down a scientific career, however, part time, at the same time as rearing children. With the passage of time, they will very likely win through on both careers, so they will have both a scientific and a genetic legacy, whereas my direct fitness will be zero!

Box 4 **Personal Reflections of Michelle Tseng, University of British Columbia, Canada. Founding Editor, Evolutionary Applications.**

The idea for a journal on applied evolutionary biology started from casual conversations I had as a graduate student with friends and colleagues who were largely unfamiliar with the many practical uses of evolutionary biology. After a comprehensive survey of the literature, I found that applied evolutionary biology papers were often published in high profile journals and were well cited, but that there was no ‘home’ journal for these types of papers. After consultation with established academic editors Loren Rieseberg and the late Harry Smith, I pitched the idea to Blackwell Publishing (now Wiley), and the journal was born. Wiley has been very supportive of *Evolutionary Applications* from the start, and I am especially grateful to Liz Ferguson at Wiley for taking a chance on this idea and for entrusting in me (at that time a recently graduated Ph.D. student) the responsibility of building the editorial team and managing the day‐to‐day operations. We implemented double‐blind reviewing from the start to help minimize biases of peer reviewers, and we consistently aimed to have a balanced gender ratio on the editorial board.

As an early‐career female in academics and academic publishing, I have been fortunate to be have been surrounded by colleagues (women and men) who have been very supportive of women in academics. Perhaps, my department is an exception, but it seems to be the norm to have a family and to excel at your career. Undoubtedly, the climate has not always been like this, and I am indebted to the many people who have fought, and continue to fight, to bring equality to the workplace. I advocate that there is room for improvement in both gender and ethnic diversity in academics. We all need to be acutely aware that our subconscious or conscious stereotypes of certain genders or ethnicities bias our ability to objectively evaluate academic achievement.

The more senior women writing in this series entered into evolutionary biology when women were still strongly dissuaded from entering science. These women speak of loved ones and teachers warning them against careers in science. For Rosemary Grant's school head mistress, mumps was evidence of ‘God's will you should not go to university’. Deborah Charlesworth writes of ‘astonishing episodes of explicit prejudice’ early on in her career. Judy Myers recalls being told by a senior scientist that ‘women don't use their Ph.D's but just get married and have babies’. Victoria Sork writes that ‘several professors advised that academia was not a place for a woman and a potential faculty mentor unabashedly informed me that he did not accept women as graduate students’. But these women's reflections also speak of persistence, resilience and a deep belief in their right to pursue a scientific career:

‘these moderate disadvantages, and the odd faculty member who tried to ignore female students, merely seemed ridiculous, and I expected them to disappear shortly (which they did)’ – Deborah Charlesworth

These senior evolutionary biologists also speak of gratitude to previous generations of women who forged a path in academia. We, in turn, are grateful to them for continuing to widen and smooth this path and for providing younger women with inspiration and role models.

Several contributors decried the fact that the very attributes that facilitate success in science are often seen as negatives traits in women. Positive descriptors including ‘*determined, motivated, persistent, stubborn, rebellious, irrepressible or independent‐minded’* become twisted when exhibited by women into negative traits such as ‘*pushy, strident, aggressive, selfish, obnoxious, mannish, unbecoming and less mentionable words’* (West‐Eberhard; Box 2). Women face accusations of being ‘too independent’ or working ‘too hard’ (Wellenreuther and Sánchez‐Guillén 2016), precisely when these qualities are also seen as critical for success.

Similarly, collaboration can be an essential element in breakthrough science, allowing a team to do what an individual cannot, yet several women voiced aggravation at automatically being given less credit than a man would have been.

‘a male scientist with a well‐developed network of collaborators is often judged to have excellent management skills, while a woman in the same position is more likely to get her independence questioned’. – Qvarnström et al. 2016.

Work done collaboratively ‘was treated as if it ‘didn't count’ towards my record, even for projects that I had initiated’ – Aktipis 2016

In this minefield where criteria for success in science mismatch gender stereotypes, several contributors provide guidance. Many women describe the usefulness of a rebellious spirit that allows you to define yourself and to forge your own path, dismissing notions of what you should be as a woman (e.g. Charlesworth 2016; West‐Eberhard in Box 2). Sometimes the road to success requires freeing yourself from those who limit you, so that you can follow your own passions. Carol Eunmi Lee, for example, describes the long and painful process of liberating herself from the limitations imposed upon her by others so that she could find and pursue her own passions and success (Lee 2016). Several authors emphasize the importance of building faith in yourself, particularly by surrounding yourself with supporters – friends and advisors – who validate your opinions, boost your confidence and expand your network (e.g. Aitken and Bemmels 2016; Johannesson 2016; Penczykowski et al. 2016; Wellenreuther and Sánchez‐Guillén 2016).

The connection between work and family was discussed in the majority of reflections. Mary Jane West‐Eberhard (Box 2) reminds us that there are some real differences that face women who seek to balance science with having children. Men do not bear, birth or breastfeed, and these and potentially other biological differences can create strong differences in family roles and commitments, both emotional and temporal. This generates, in her view, ‘the central problem for women in science’ of how to ‘harmonize relationships and family life with a full‐blown scientific career’. She writes of the many creative, if extreme, ways in which our colleagues have done so.

Several contributors with children expressed positive opinions about being both a parent and a scientist that suggest that we need to reframe the concept of ‘work–life balance’. Often this phrase is used to emphasize an inherent (negative) trade‐off between time spent doing science and time spent on other pursuits, including child rearing. But many authors did not see it this way. Women spoke of a different, more positive interpretation of this balance: the advantage of a life that is not overly focused and the benefits of stepping back occasionally from work to see the broader picture. Raising children can enforce this balance.

‘I truly believe that my two children make me a better scientist. Motherhood has helped me recognize my priorities and manage my time. Also, there is no better way of decompressing after work than being with kids, as they demand 100% of your attention’. – Britt Koskella (2016)

‘I believe that working fewer hours can actually benefit research, so long as the actual working hours allow time for thinking – the ideas then sometimes mature in the “non‐working” hours’. – Deborah Charlesworth (2016)

The reverse point was also made that children benefit from exposure to their parents’ work, especially when fieldwork accommodates children (Charmantier et al. 2016; Grant in Box 1; Gillespie 2016). The work–life balance should thus be framed as finding your personal optimum: the combination of work, sports, music, art, family and/or adventure that personally makes you content and that allows you to work to the best of your ability. It is also important to remember not to let life pass too fast. If you want to make sure that you reach old age having a particular experience, then do not repeatedly put off that experience for the sake of work. Decide when to do it and make it happen.

Many women also emphasized the many positives of being a scientist, particularly the freedom, stimulation and deep satisfaction that comes from discovery. The freedom is truly wonderful – we can choose what to study, where and when. Academia also allows more freedom than most jobs to alternate time at home and at work, as needed. Indeed, the ability to strike one's own balance is a major perk, as Victoria Sork notes: ‘I cannot imagine a career that is more family‐friendly than being a university professor’.

The life‐long intellectual stimulation from students, collaborators and research discovery is another tremendous privilege. Johannesson (2016) urges us to remember ‘that the most important driver in research is to have fun at work’, and many contributors expressed how much they love what they do. The rewards of collaboration and working as a part of a team were also frequently raised.

‘This is our experience of research, as female scientists at various stages of their career: we feel very lucky having worked in an environment where cooperation was always valued above competition, and where friendship was intricately mixed with high intellectual stimulation’. – Olivieri et al. (2016)

## Ways forward

The contributions in this special issue also provide recommendations to improve the lives of scientists in ways that will encourage women to enter science, stay in science and help repair the leaky pipeline. A recurrent theme is the need for more supportive policies for academic parents, which can include parental leave, at‐work day care, help with care for sick children, travel support for young children, day care at conferences, part‐time early‐career appointments and policies that smooth the return to science following parental leave. As emphasized by Susan Haig (2016), ‘it is clear that as a profession, we have work to do to address lifestyle demands or we risk losing the best and brightest researchers and laboratory workers’.

Women benefit from learning from each other how to navigate family life and work–life. One way forward is to develop improved university‐wide policies that pool together the best of the practices in various departments – learning also from forward‐thinking policies at other Universities; this especially benefits women isolated in male‐dominated departments. As an example of best practices, it has been suggested that reviewers of job and grant applications should use the ‘academic’, rather than chronological, age when it comes to the evaluation of applicants to avoid penalizing women who have taken parental leave (Lane 1999).

Many women emphasized the benefits of networking, to open doors to new opportunities as well as to build the circle of collaborators and advisors who will support you in your career. Several caution against the time demands of administration and service tasks. This issue is particularly acute for women, as Universities, granting agencies, and journals strive for gender balanced committees even though the source pool (especially among senior academics) remains imbalanced. This pull towards service can limit research time and research success, and so you should evaluate carefully requests for service (e.g. setting a fair time budget for such service and sticking to it).

Other advice describes how to lessen implicit biases. For example, Leftwich et al. (2016) suggest a greater reliance on computer‐based citation searches to develop references for a paper, rather than relying solely on memory, which is more susceptible to bias. Strategies also exist to reduce bias in publishing itself, which have been described by Budden et al. (2008): Keeping authors of scientific papers anonymous has been shown to improve women's odds of acceptance. The practice can also block bias against minorities and bias in favour of authors and institutions with big reputations. Double‐blind peer review, in which the identities of both authors and reviewers are hidden from one another, is already common in the social sciences and humanities but still rarely practiced in ecology or evolution journals. As highlighted by Tseng (Box 4), Evolutionary Applications has been a pioneer in the field by adopting a double‐blind strategy since its foundation in 2008, with the aim to avoid our implicit biases and to ensure the fair assessment of research quality. Cognitive biases are so numerous and universal that, at the very least, we should make ourselves aware of how deep they can run. For faster change, we all need to act as exemplars – correcting our own mistakes, and monitoring biases around us. The stakes are higher than most of us realize. Biases in academia stifle the insight we could be gaining from a more diverse set of collaborators.

Indeed, Pemberton (Box 3) points out that the number of women on corporate boards has been shown to be positively correlated with business success (e.g. profits). She suggests that we investigate and document the ways in which progress in science is also advanced by having more diversity in sciences. Such evidence would provide further incentive to stop the leaky pipeline and promote gender equality.

In closing, this issue is an exciting opportunity to paint a new portrait of the changing face of evolutionary biology, as illustrated by our front cover. Women are altering the way we think about evolutionary biology, charting new research directions, smoothing the path for other women, and loving their jobs. Obstacles and hiccups remain, but there also are incredible opportunities for women entering the field. To young women considering a career in academia, find your passion in research, have faith in yourself (or construct it if needed), and strive to accomplish all that you can accomplish. As obscure as the path can seem, the journey is deeply satisfying.

‘love what you do with a passion ‐ and do what you love with equal passion’ – Rosemary Gillespie (2016)

## Notes

1
http://www.inserm.fr/mediatheque/infr-grand-public/fichiers/l-ethique-a-l-inserm/affaire-marthe-gautier-et-regles-publication.pdf.

2
http://www.interacademies.net/Academies/UnitedStates/13155.aspx.

3
http://www.awis.org/general/custom.asp?page=Awards_NAS.

4
http://www.nobelprize.org/nobel_prizes/facts/.

5
http://www.evolutionsociety.org/content/society-awards-and-prizes/the-theodosius-dobzhansky-prize.html.
